# Development and application of EpitopeScan, a Python3 toolset for mutation tracking in SARS-CoV-2 immunogenic epitopes

**DOI:** 10.3389/fimmu.2024.1356314

**Published:** 2024-05-22

**Authors:** Alexander Kovalenko, Sebastien Viatte

**Affiliations:** ^1^ Versus Arthritis Centre for Genetics and Genomics, Centre for Musculoskeletal Research, Manchester Academic Health Science Centre, The University of Manchester, Manchester, United Kingdom; ^2^ Lydia Becker Institute of Immunology and Inflammation, Faculty of Biology, Medicine and Health, The University of Manchester, Manchester, United Kingdom; ^3^ National Institute for Health and Care Research (NIHR) Manchester Musculoskeletal Biomedical Research Centre, Central Manchester National Health Service (NHS) Foundation Trust, Manchester Academic Health Science Centre, Manchester, United Kingdom

**Keywords:** SARS-CoV-2, epitope, mutation tracking, vaccine design, rheumatoid arthritis, immune monitoring, software

## Abstract

**Introduction:**

Outbreaks of coronaviruses and especially the recent COVID-19 pandemic emphasize the importance of immunological research in this area to mitigate the effect of future incidents. Bioinformatics approaches are capable of providing multisided insights from virus sequencing data, although currently available software options are not entirely suitable for a specific task of mutation surveillance within immunogenic epitopes of SARS-CoV-2.

**Method:**

Here, we describe the development of a mutation tracker, EpitopeScan, a Python3 package with command line and graphical user interface tools facilitating the investigation of the mutation dynamics in SARS-CoV-2 epitopes via analysis of multiple-sequence alignments of genomes over time. We provide an application case by examining three Spike protein-derived immunodominant CD4^+^ T-cell epitopes restricted by HLA-DRB1*04:01, an allele strongly associated with susceptibility to rheumatoid arthritis (RA). Mutations in these peptides are relevant for immune monitoring of CD4^+^ T-cell responses against SARS-CoV-2 spike protein in patients with RA. The analysis focused on 2.3 million SARS-CoV-2 genomes sampled in England.

**Results:**

We detail cases of epitope conservation over time, partial loss of conservation, and complete divergence from the wild type following the emergence of the N969K Omicron-specific mutation in November 2021. The wild type and the mutated peptide represent potential candidates to monitor variant-specific CD4^+^ T-cell responses. EpitopeScan is available via GitHub repository https://github.com/Aleksandr-biochem/EpitopeScan.

## 1 Introduction

Coronaviruses (CoVs) are enveloped RNA viruses mainly infecting vertebrates, with species of *Alpha-* and *Betacoronavirus* genera also being known as zoonotic human pathogens commonly causing mild respiratory diseases (1). Since the beginning of the century, reoccurring zoonotic spillovers of *Betacoronaviruses* posed a more serious threat to public health. Namely, these are the SARS-CoV outbreak in China in 2003 (8,096 cases registered, 774 lethal, case fatality rate ~10%) (2) and emergence of Middle Eastern respiratory syndrome virus (MERS-CoV) in 2012 followed by its spread among countries of Middle East, South Asia, and Africa (2,494 cases registered, 858 lethal, case fatality rate 35%) (3). The most recent and notorious case is SARS-CoV-2, the causing agent of the COVID-19 disease, which emerged in Hubei province of China in late 2019 (4). The outbreak rapidly escalated to a pandemic, as declared by WHO on 11th March 2020 (5). During the pandemic, more than 676 million cases with over 6.8 million related deaths have been recorded around the world (as reported of March 2023) (6), with a case fatality rate ranging from 0.1% to 4.9% depending on the region (7). COVID-19 remains a current healthcare issue, and there are questions yet to be answered regarding the threat posed by the emergence of new variants and the longevity of variant-specific immunity, as well as about causes and effects of long COVID (8). Moreover, there is a definite threat of future zoonotic spillovers of CoVs (9). Therefore, a detailed research on biological and clinical aspects of SARS-CoV-2 remains an important objective in preparation for any similar incidents.

Methods to determine the magnitude and longevity of CD8^+^ and CD4^+^ T-cell responses specific for a viral peptide of interest include the use of fluorophore-labelled, peptide-loaded HLA-class I and class II tetramers (for CD8^+^ and CD4^+^ T cells, respectively). *Ex vivo*, peptide-loaded tetramers will bind specifically to the T-cell receptor (TCR) of T cells, which have the capacity to specifically recognize the peptide of interest *in vivo* (10). T cells stained by tetramers are amenable to be detected, enumerated, phenotyped, and monitored by flow cytometry (a single-cell technique using a set of lasers to detect fluorophore-labelled cells). For example, this powerful technique allows the monitoring of T-cell responses specifically directed against individual peptides derived from the Spike (S) protein of SARS-CoV and SARS-CoV-2. Immunodominant S protein epitopes, fully conserved between these two CoVs, were already identified before the COVID-19 pandemic (11). We refer to three immunodominant CD4^+^ T epitopes restricted by the HLA class II allele HLA-DRB1*04:01 and conserved between SARS-CoV and the original Wuhan variant of SARS-CoV-2 as PS1, PS2, and PS3 peptides. Tetramers loaded with these peptides have the capacity to detect cross-reactive CD4^+^ T-cell responses directed against both viruses, but their use to monitor CD4^+^ T-cell immunity against SARS-CoV-2 over time requires experimenters to know which peptide is affected by mutations over time. Notoriously, new SARS-CoV-2 variants appear regularly over the course of the pandemic, and mutations within PS1, PS2, or PS3 could represent immune escape mechanisms, disrupting peptide binding to HLA molecules, or, if not, leading to a loss of recognition by TCRs.

Bioinformatics approaches coupled to rich genomic data proved invaluable to SARS-CoV-2 research and pandemic monitoring (12). When it comes to immunological aspects, sequence analysis can provide valuable insights into conservation of immunogenic epitopes and the effects of mutations on immune response. Several tools for SARS-CoV-2 mutation surveillance are available. Online resources such as GISAID EpiCoV™ (13), COG-UK Mutation Explorer (focused on the data from the United Kingdom) (14), COVID CG (15), and CoV-GLUE (16) accumulate data from across the world to generate informative statistics on mutation. With regard to immunity-related surveillance, the T-CoV online atlas provides a database linking most frequent mutations to their effect on immunogenic peptides’ interactions with HLA class I or II (17). These online tools provide a general overview from the analysis of large genome sets encompassing multiple populations. It may be relevant to study custom sets of genomes limited to smaller subpopulations of interest. Such analysis could be performed with online GISAID CoVsurver (13), although it would require a user to perform a manual processing of the output to extract information on the epitopes of interest. Analysis of mutation within particular protein regions can also be addressed in a standalone manner with custom pipelines combining software for variant calling and annotation. This strategy requires a certain level of proficiency in bioinformatics. Alternatively, there are tailored software options that are less demanding in terms of specialized knowledge. For example, immunologists may benefit from a semiautomated COVID-miner Python3 and R pipeline, which performs mutation frequency analysis and builds a consensus sequence for vaccine design (18). This pipeline provides limited versatility for custom analyses, especially to focus on panels of distinct epitopes rather than on full-length proteins. Another Python 3 package, MicroGMT, is designed to summarize mutations in SARS-CoV-2 sequences and other viral or microbial species (19). Although one could use MicroGMT to filter data on mutations in distinct genome regions, it provides no tools for subsequent analysis of mutation frequency and dynamics in a manner relevant to immunological research.

With the aim to bridge this gap, we developed a new software, EpitopeScan, for a customizable analysis of mutations in SARS-CoV-2 immunogenic epitopes from genome alignment data. EpitopeScan is a Python3 package providing a command line and graphical interfaces for a streamlined multisided analysis of epitopes originating from any SARS-CoV-2 protein. **Table 1** compares the features and scopes between EpitopeScan and other tools mentioned above. We provide an application case of EpitopeScan by analyzing three experimentally characterized CD4^+^ T-cell epitopes from the S protein (PS1, PS2, PS3). Since these epitopes are restricted by HLA-DRB1*04:01, one of the HLA class II alleles most strongly associated with rheumatoid arthritis (RA) (20), mutation tracking over time allows to investigate whether these peptides are suitable for immune monitoring of SARS-CoV-2-specific CD4^+^ T-cell responses at a specific point in time (post-vaccination or postinfection) in RA patients bearing at least one copy of the HLA-DRB1*04:01 allele. This case demonstrates how EpitopeScan can be helpful in investigation of the magnitude and longevity of variant-specific immunity in vulnerable patients with autoimmune diseases under immunosuppressive treatment, which represents an unmet clinical need.

**Table 1:** A comparison of the features between tools and resources, which can be used to track mutations in SARS-CoV-2 and assess their influence on immunogenic peptide epitopes.

Tool/resource	Implementation	Input data	Features relevant for immunological research	Limitations	Graphical user interface
GISAID EpiCoV™	Online	Central repository of SARS-CoV-2 genomes	Lineage frequency and variant analysis	Works mainly as a sequence database with no focus on peptide epitopes	Yes
COG-UK Mutation Explorer	Online	COG-UK database of SARS-CoV-2 genomes	Incorporates T-cell epitope mutation database and browser	Operates only on data collected in the UK. No means to analyze custom peptides absent in the database	Yes
COVID CG	Online	GISAID repository of SARS-CoV-2 genomes	Lineage and protein mutation reports	Works mainly as a mutation database with no focus on peptide epitopes	Yes
T-CoV	Online	GISAID repository of SARS-CoV-2 genomes	Data on mutations in CD8^+^ and CD4^+^ epitopes by viral variant	No means to analyze custom peptides of interest and regional frequency of mutation	Yes
CoVsurver	Online	User-supplied genome or protein sequences	Lists mutations present in a custom set of sequences	No direct focus on peptide epitopes’ statistics	Yes
CoV-GLUE	Online	GISAID repository of SARS-CoV-2 genomes	Lineage and protein mutation reports by country	No direct focus on peptide epitopes’ statistics	Yes
MicroGMT	Standalone (Python3)	User-supplied raw sequence reads or genome sequences	Lists mutations present in a custom set of sequences	No direct focus on peptide epitopes’ statistics	No
COVID-miner	Standalone (Python3, R)	GISAID data or user-supplied sequences	Consensus sequence and mutation analysis facilitating vaccine design	No direct focus on peptide epitopes’ statistics	No
**EpitopeScan**	Standalone (Python3)	User-supplied genome alignment and peptide epitope sequences of interest	Mutation reports tailored to analyze conservation of immunogenic peptides	Requires prealigned sequences	Yes, for mutation statistics analysis

## 2 Method

### 2.1 EpitopeScan package implementation

#### 2.1.1 EpitopeScan components and general workflow

In this work, we developed EpitopeScan, Python3 package facilitating mutation tracking in SARS-CoV-2 protein sequences with the focus on user-defined regions of interest, such as immunogenic peptides. EpitopeScan is freely available via the GitHub repository with a detailed manual and usage examples (https://github.com/Aleksandr-biochem/EpitopeScan). The main package components are:

- EpitopeScan.py Command-Line Tool (CLT) for mutation report from genome Multiple Sequence Alignment (MSA). CLT operates in either scan mode (generating mutation data on peptides of interests from supplied genomes), or stat mode (generating statistics from preexisting scan output).
- EpitopeScanGUI.py Graphical User Interface (GUI) application built with Streamlit 1.20.0 and Plotly 5.14.0 Python libraries. The application uses system’s web-browser interface to provide an interactive report from mutation data generated by CLT.

Internal reference data for the package were collected from the GISAID “Official hCoV-19 Reference Sequence” resource in January 2023 (21). It includes SARS-CoV-2 genome (isolate WIV04, GISAID accession code EPI_ISL_402124) (22), genome coordinates for 15 open reading frames (ORFs), and reference protein sequences (30 sequences).

**Figure 1:**
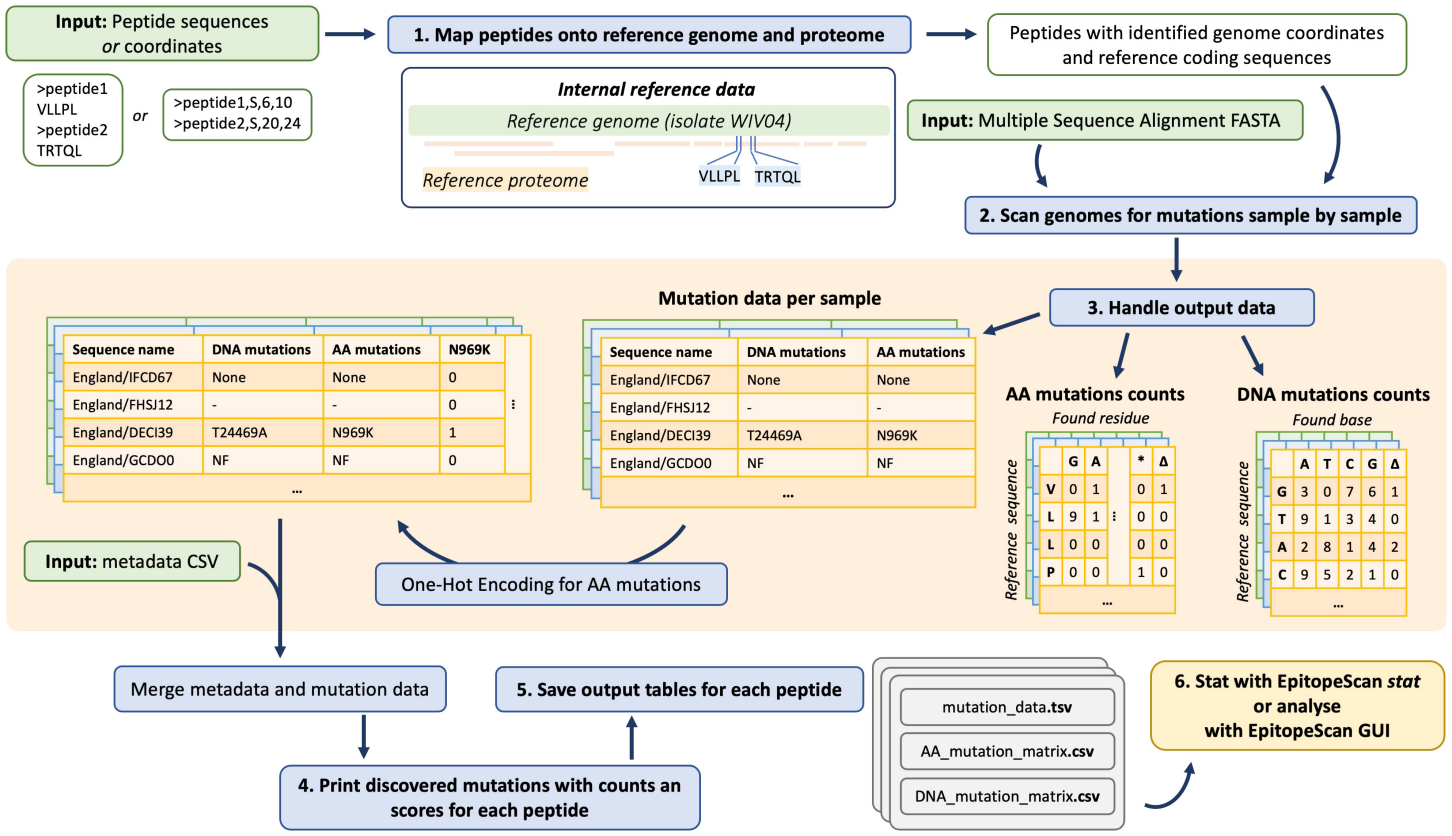
EpitopeScan analysis workflow. Input peptides are supplied as sequences or residue spans in parent proteins. First, peptides are mapped onto reference proteome and genome to determine coordinates to focus on during MSA processing. Genomes from MSA are processed sequentially. For each peptide, three tables are generated: DNA and amino acid (AA) mutation count matrices and a table with mutation data per sample. The latter table is expanded using one-hot encoding for discovered AA mutations. These mutation data are merged with sample metadata (if provided). Finally, the mutation summary is printed into terminal output and the output tables are saved on the system for subsequent analysis with EpitopeScan stat, EpitopeScan GUI, or any other software.

EpitopeScan analysis workflow is summarized at **Figure 1**. The required inputs are SARS-CoV-2 peptides of interest and genome MSA in FASTA format. The tool does not perform genome alignment; therefore, the genomes should be aligned separately before analysis using the EPI_ISL_402124 sequence as reference. An additional recommended input is metadata on samples’ dates and assigned lineages, which allows to get more insights into dynamics of mutation spread. Mutation data produced by CLT can be summarized and investigated using the CLT stat mode, GUI application, or any other software (for example, R).

#### 2.1.2 Peptide handling and mapping onto the reference genome

Peptides for the analysis can be provided either as sequences or as residue spans in proteins, from which these peptides originate (further termed as parent proteins). For example, “VLLPL” sequence input is equal to “S,6,10” (residues 6 to 10 in S protein). Residue span input allows to analyze short peptides, which map ambiguously onto proteome. Input peptides and reference SARS-CoV-2 proteins are handled as “Protein” class instances implemented in the package. “Protein” class attributes are the object’s name, protein sequence, reference DNA coding sequence, genome start and end coordinates, DNA and amino acid (AA) substitution matrices, names of parent proteins, and the start coordinate in the main parent protein (for peptides, which are subsequences of the other proteins).

This structure facilitates mapping of the input peptides onto reference proteins. The mapping step defines genome coordinates to focus on during sequence analysis (**Figure 1**, step no. 1). Protein class methods and package functions account for a –1 frame shift in the SARS-CoV-2 Open Reading Frame 1ab (ORF1ab) at the genome position 13,469. Some SARS-CoV-2 ORFs are nested (ORF1a and 1b lie within ORF1ab, ORF9a, and 9b within N), and the ORF1ab polyprotein (Plp1ab) produces multiple non-structural proteins (NSPs). In order to deal with such structure, EpitopeScan picks the largest protein as the primary parent to define peptide coordinates while also reporting relation to other proteins. For example, a peptide may be attributed to Plp1ab with protein start coordinate defined accordingly, while also listing relation to Plp1a and NSP3.

#### 2.1.3 Sequence processing and mutation report

**Figure 2:**
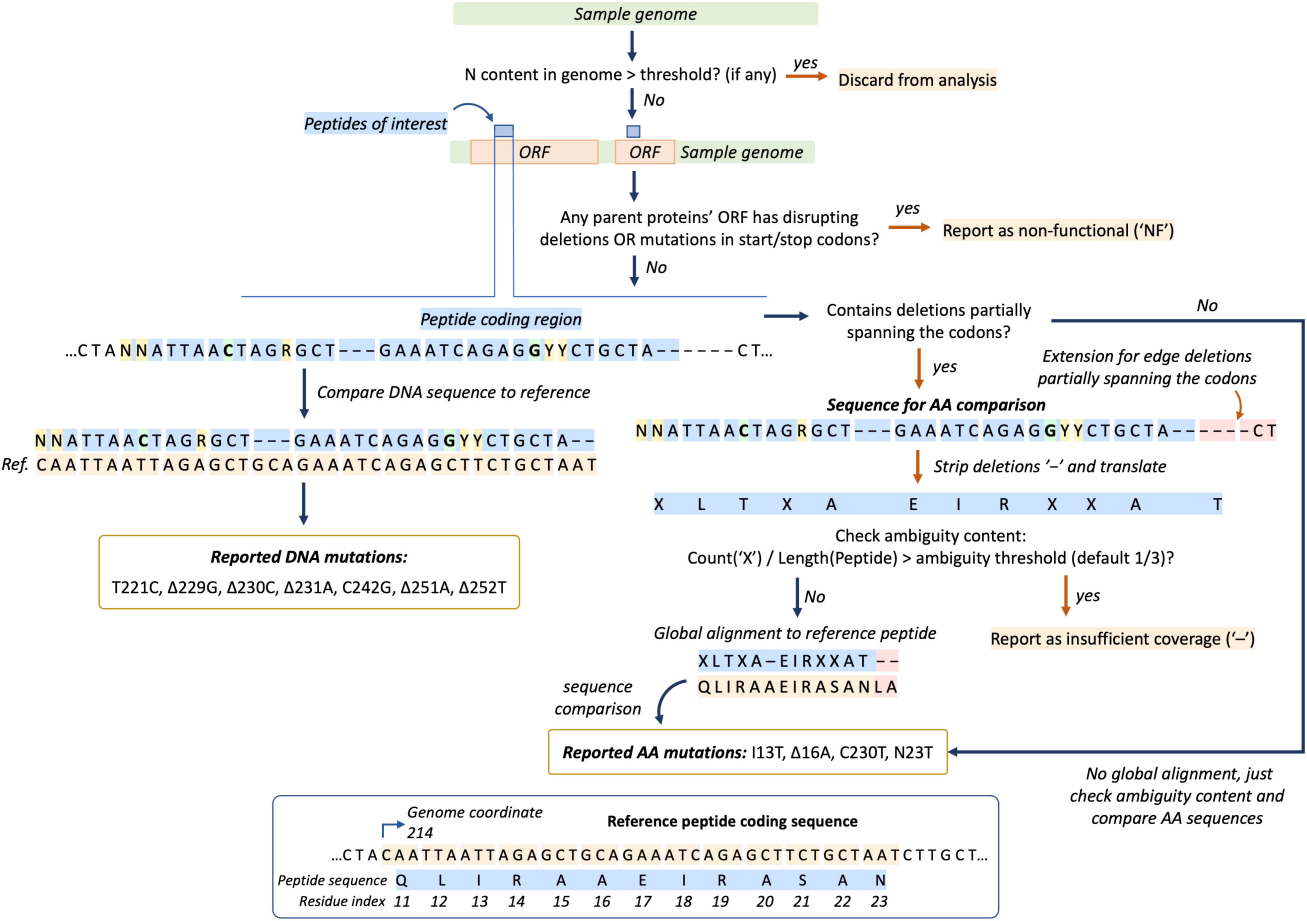
Genome sequence (green bar) processing algorithm in EpitopeScan. First, the “N” base content filter is applied (if any). Next, peptides’ (blue bars) parent proteins ORFs (orange bars) are scanned for frame-disrupting mutations and start/stop codon mutations. If any are found, the sample is marked as non-functional (“NF”). Then, the DNA sequence region corresponding to a peptide is directly compared with reference DNA sequence to report DNA mutations (branch on the left). Amino acid (AA) mutations are reported separately by comparing translated sequences (branch on the right, “X” stands for ambiguous residues). The panel at the bottom illustrates reference peptide and coding sequences.

Sequences from genome MSA are processed through steps illustrated at **Figure 2**. If specified, the genome quality filter based on ambiguous “N” base content is applied. The proportion of “N” bases is calculated as the number of “N” bases in a genome divided by the length of the genome after stripping deletion symbols (“-”). When the proportion is larger than a user-defined threshold, the sequence is discarded from analysis.

Prior to mutation calling, the algorithm searches parent protein ORF for deletions disrupting translation. Any in-frame deletion with the number of positions not equal to a multiple of 3 leads to disrupted translation (see visualization at **Supplementary Figure 1**). Such samples are marked as non-functional (“NF” in output table). Mutations in start or stop codons of the parent protein ORF also lead to “NF” report. Nonsense mutations inside ORFs are reported explicitly (for example, Q903*).

If no disrupting deletions are found, the peptide coding sequence is compared with the reference to report DNA substitutions and deletions. AA mutations are reported separately by comparison between translated DNA sequences. Only precisely resolved bases are recognized in sequence comparison. Therefore, for a codon like ‘CAR’ (R is A or G) with reference ‘CAC’ no DNA or AA mutation will be reported, even though technically it corresponds to substitution from histidine to glycine. In addition, for an ambiguous codon ‘GGY’ (Y is C or T) with reference ‘GCT’, C to G substitution will be reported without any AA substitution (**Figure 2**).

In case the DNA sequence contains deletions partially spanning the codons, translated peptide sequences are prealigned using the Needleman–Wunsch algorithm allowing alignment-based report of AA substitutions and deletions (**Figure 2**). In case partially spanning deletions are situated at the edges of coding sequence, the sample and reference DNA sequences are extended to capture additional length (**Figure 2**). Sequences with proportion of ambiguous codons exceeding the threshold (default one third) are reported with insufficient coverage (“-” in output table, **Figure 2**).

As a result, three tables are generated for each peptide: per sample mutation data, AA and DNA substitution matrices (**Figure 1**, middle orange panel). Substitution matrices hold counts of DNA bases (or AA residues) and deletions found at each position of the reference DNA and AA sequences respectively. These counts are accumulated only from functional samples with sufficient coverage. The mutation data table contains sample names, two columns listing DNA and AA mutations, followed by columns indicating presence of discovered AA mutations using one-hot encoding.

### 2.2 EpitopeScan mutation report testing

The EpitopeScan algorithm for the mutation report was tested against 31 peptides covering each SARS-CoV-2 protein (sequences listed in **Supplementary Table 1**). For each peptide, 1,000 genomes with reference mutation data were simulated by randomly introducing mutations to the corresponding regions of the reference sequence. The simulated sequences feature various cases of substitutions, deletions, ORF disruption, insufficient coverage, and high “N” base content. Test data were prepared with an in-house Python3 notebook, which is available in **Supplementary Materials**. Test peptides’ data and the testing script are included with the EpitopeScan package on GitHub.

In addition, the EpitopeScan mutation report was verified against 2,334,011 SARS-CoV-2 genomes sampled across the United Kingdom (UK) with metadata listing mutations for each sample. The data were obtained from the COVID-19 Genomics UK Consortium (COG-UK) public data resource (https://www.cogconsortium.uk/priority-areas/data-linkage-analysis/public-data-analysis/, accessed 1st June 2023). EpitopeScan mutation data were generated for three peptides from Spike protein (PS1–3, **Table 2**) at default CLT options. Comparison with COG-UK mutation metadata was performed with the in-house Python notebook, which is available in **Supplementary Materials**.

**Table 2:** Key information on three SARS-CoV-2 Spike (S) protein-derived peptides PS1–3 (sequence, coordinates in S protein and coordinates in the reference genome).

Peptide:	PS1	PS2	PS3
Sequence:	MAYRFNGIGVTQNVLY	QALNTLVKQLSSNFGAI	QLIRAAEIRASANLAATK
S protein residues:	902–917	957–973	1,011–1,028
Genome coordinates:	24,266–24,313	2,4431–24,481	24,593–24,646
Have mutations	1,092 (0.05%)	1,083,431 (46.42%)	9,200 (0.39%)
No mutations	2,323,356 (99.54%)	1,242,561 (53.24%)	2,317,022 (99.27%)
Insufficient coverage	3,795 (0.16%)	2,251 (0.10%)	2,021 (0.09%)
Non-functional	5,768 (0.25%)	5,768 (0.25%)	5,768 (0.25%)

General sample categories (counts and percentage in all samples) from the EpitopeScan analysis report calculated from 2,334,011 samples with metadata. “Insufficient coverage” refers to samples with at least one ambiguous base in a corresponding peptide coding region. “Non-functional” refers to samples with disrupting deletions or mutations in start/stop codons of Spike protein Open Reading Frame.

EpitopeScan performance was assessed for varying lengths of input peptide (S protein peptides of 5, 10, 15, 20, 30, and 50 residues) and for varying numbers of input peptides (1, 3, 6, 10, and 20 peptides of five residues in length). EpitopeScan runs were conducted on 3,392,463 genomes sampled across the UK (obtained from COG-UK public data resource on 1st June 2023), and the run times were recorded with standard terminal time utility. Peptide inputs and bash scripts from this step are available with **Supplementary Materials**.

### 2.3 Analysis of mutations in SARS-CoV-2 Spike protein peptides

EpitopeScan was applied to study mutation in three Spike protein peptides known to be CD4^+^ T-cell-specific epitopes restricted by the HLA-DRB1*04:01 allele (PS1–3, **Table 2**). SARS-CoV-2 data for analysis was obtained from the COG-UK public data resource and included unmasked MSA of 3,392,463 genomes sampled across the UK between 29/01/2020 and 17/05/2023, and the metadata table for 2,995,815 UK samples (accessed 1st June 2023). Only samples from England were kept for the analysis. Three EpitopeScan CLT analysis configurations were compared: (i) default options; (ii) ambiguity threshold of 0 (presence of any ambiguous base in the peptide coding sequence is treated as insufficient coverage); (iii) ambiguity threshold of 0 and “N” base content filter with the upper threshold of 0.05 (5%). Runs were launched with the following commands:

- $./EpitopeScan.py scan -f path/to/epitopes.fasta –msa path/to/cog_unmasked_alignment.fasta–metadata path/to/cog_metadata.csv -t England
- [command (i)] –ambiguity_threshold 0.0
- [command (i)] –ambiguity_threshold 0.0 –quality_filter 0.05

The EpitopeScan CLT stat mode was applied to calculate general sample statistics and mutation counts. Mutation data from run (ii) were subjected to a detailed analysis of peptides’ mutation history in EpitopeScan GUI. These data are also available in **Supplementary Materials**. History of SARS-CoV-2 lineage spread in England was researched with the Wellcome Sanger Institute’s (WSI) geographical lineage prevalence resource (23). MHC II restriction for PS1–3 and PS2 with the N969K mutation was estimated with the T-cell epitope MHC II binding prediction online tool (IEDB Analysis Resource v2.26) (24). All peptides were processed at once specifying the HLA-DRB1*04:01 allele, full-length peptide processing, and IEDB 2.22 method, which generates a consensus prediction from the SMM-align (25) and NN-align (26) tools and the Sturniolo method (27).

## 3 Results

### 3.1 EpitopeScan performs streamlined meaningful mutation report and facilitates output analysis

The EpitopeScan package was developed to provide a user-friendly workflow facilitating generation and interpretation of mutation data from SARS-CoV-2 genome MSA with the focus on peptides of interest. EpitopeScan CLT is equipped with multiple flags to tune sample filtering by name patterns and quality thresholds (“N” base content in genome and ambiguity content in the peptide coding region). The CLT stat mode with adjustable report options provides a quick overview of sample categories with mutation counts. Mutations can be counted as individual species or as occurring combinations. In addition, for each mutation or combination, a corresponding BLOSUM score is displayed. BLOSUM scores add an extra dimension to the report, as they provide an estimate of how likely a mutation is to appear in a biological sense (28). Further mutation data analysis is supported by EpitopeScan GUI, which employs metadata on sample dates and lineages to generate time plots on sample categories and mutation counts, and to provide insights into propagation of mutations in population.

Two controls for the correctness of mutation report in EpitopeScan were included in the study: the randomly simulated data and the COG-UK genome sequences with mutation metadata. The algorithm takes into consideration tunable sequence quality requirements and loss of functionality due to ORF disruption, which provides an additional quality control point and is not accounted for in COG-UK metadata. In terms of performance, EpitopeScan scan completes the analysis of a Spike protein peptide PS1 (16 residues, **Table 2**) against 3.39 million genomes within 14 min on 1 CPU (Apple M1 chip 2020) with RAM uptake up to 10 GB. The run time increases exponentially with input peptide length reaching 4.5 h for a test S protein peptide of 50 residues (detailed data provided at **Supplementary Table 2** and **Supplementary Figure 2**). Dependence on the number of input peptides is not as straightforward. ORF control for disruptions appears to be the time-limiting step, as the run time was found to be exponentially related to the cumulative length of parent protein ORFs (detailed data provided at **Supplementary Table 3** and **Supplementary Figure 3**).

### 3.2 Analysis of mutation in three Spike protein epitopes details varying mutation history

In order to demonstrate EpitopeScan utility, we conducted a mutation analysis for three peptides PS1–3 from the S protein of SARS-CoV-2. **Table 2** provides key information on these peptides including their sequences, coordinates in S protein, and the reference genome (as identified by EpitopeScan peptide mapping). Genome MSA employed for the analysis contains 2,671,810 samples from England dated between 29/01/2020 and 17/05/2023. The number of England’s samples supported by metadata on date and viral lineage is 2,334,011 (87.4% of all). The following plots and statistics are generated only from the samples with metadata. The focus on England’s samples allows application of the WSI geographical lineage prevalence resource to track spread of lineages related to certain mutations in PS1–3.

Mutation data generated from EpitopeScan run with an ambiguity threshold of 0 were utilized for a detailed mutation tracking in EpitopeScan GUI. Compared with other tested analysis configurations (see 2.3 Methods), this one allowed to preserve more samples while imposing stricter requirements on sequence quality in PS1–3 coding regions (see detailed statistics comparison in **Supplementary Table 4**). **Table 2** lists key sample categories for each peptide generated with the CLT stat mode. Judging from these general statistics, PS2 appears to be least conserved with almost half of the samples featuring some mutations. In contrast, PS1 and PS3 are overall well-conserved, although it is notable that proportion of PS3 samples with mutations is approximately eight times higher than that of PS1.

Mutation counts for PS1–3 (**Table 3**) generated with EpitopeScan stat mode highlight additional details to the general sample statistics. No cases of cooccurring mutations with significant counts were found (“combinations” in **Table 3**, counts not shown); therefore, all mutations were investigated as independent species. Overall, only few individual mutations have high counts of >300–1,000, whereas the vast majority have counts <10. According to **Table 3**, PS1 has the least number of mutations emerging throughout the pandemic. Even the most abundant mutation I909V has rather low count compared with the overall number of samples. PS1 generally features mutations with positive BLOSUM scores, suggesting that this region is prone to likely substitutions, which are not propagating in population. PS2 features the N969K mutation, which is the most abundant one across the studied peptides. It is present in roughly half of all samples, making it the likely cause of conservation loss in PS2. This mutation is also present in the vast majority of samples dated in recent months (**Table 3**), indicating its prevalence in circulating lineages. In addition, EpitopeScan stat counts for combined mutations highlight that all mutations emerging in recent months of 2023 appear in combinations with N969K (data not shown). All of the other mutations of PS2 have significantly lower counts. PS3 displays the highest number of individual mutations throughout the pandemic (average 1.48 mutations per PS3 residue compared with 1.08 for PS2), although the majority of them have low counts below 10–100 and do not form as much combinations as PS2. Therefore, PS3 mutation seems to be overall stochastic despite its abundance. Nevertheless, mutations A1020S and A1020V may be of interest for a detailed investigation as their counts are moderately high.

**Table 3:** Mutation counts for PS1–3 peptides generated with the EpitopeScan stat in three time periods: throughout the whole pandemic, January–May 2023, and in the last week of the monitored period.

Dates, y/m/d	2020/01/29–2023/05/17									
N samples	2,333,823									
Peptide	PS1			PS2			PS3			
Individual mutations	32			43			69			
Combinations	32			64			75			
	*Mutation*	*Count*	*Score*	*Mutation*	*Count*	*Score*	*Mutation*	*Count*	*Score*
	**I909V**	384	3.0	**N969K**	1082187	0.0	**A1020S**	5152	1.0
	V911I	174	3.0	**Q957L**	580	-3.0	**A1020V**	1452	-1.0
	Q913H	146	1.0	**T961M**	496	−1.0	T1027I	976	−1.0
	V915I	146	3.0	A958S	116	1.0	L1024F	210	0.0
	A903S	52	1.0	T961A	53	0.0	A1016V	194	−1.0
Dates, y/m/d:	2023/01/01–2023/05/17									
N samples:	48,850									
Peptide:	**PS1**			**PS2**			**PS3**			
Individual mutations	4			8			14			
Combinations	4			8			14			
	*Mutation*	*Count*	*Score*	*Mutation*	*Count*	*Score*	*Mutation*	*Count*	*Score*
	**I909V**	6	3.0	**N969K**	48,661	0.0	**A1020S**	30	1.0
	V911I	4	3.0	T961M	23	−1.0	**A1020V**	17	−1.0
	V915I	2	3.0	V963I	4	3.0	A1016S	6	1.0
	Q913H	1	1.0	Q957L	4	−3.0	N1023S	3	0.0
	–	–	–	S967G	2	−1.0	N1023I	3	−4.0
Dates, y/m/d:	2023/05/10–2023/05/17									
N samples:	377									
Peptide:	**PS1**			**PS2**			**PS3**			
Individual mutations	0			3			0			
Combinations	0			3			0			
	–			*Mutation*	*Count*	*Score*	–	–	–
	–			**N969K**	377	0.0	–	–	–
	–			S967G	1	−1.0	–	–	–
	–			K964E	1	0.0	–	–	–

For each period, the number of samples is specified. For each peptide, there is a number of individual mutations registered in each period and the number of unique combinations of mutations occurring in these samples. These data are followed by counts and BLOSUM scores for the five most occurring individual mutations of that period (if any).

**Figure 3:**
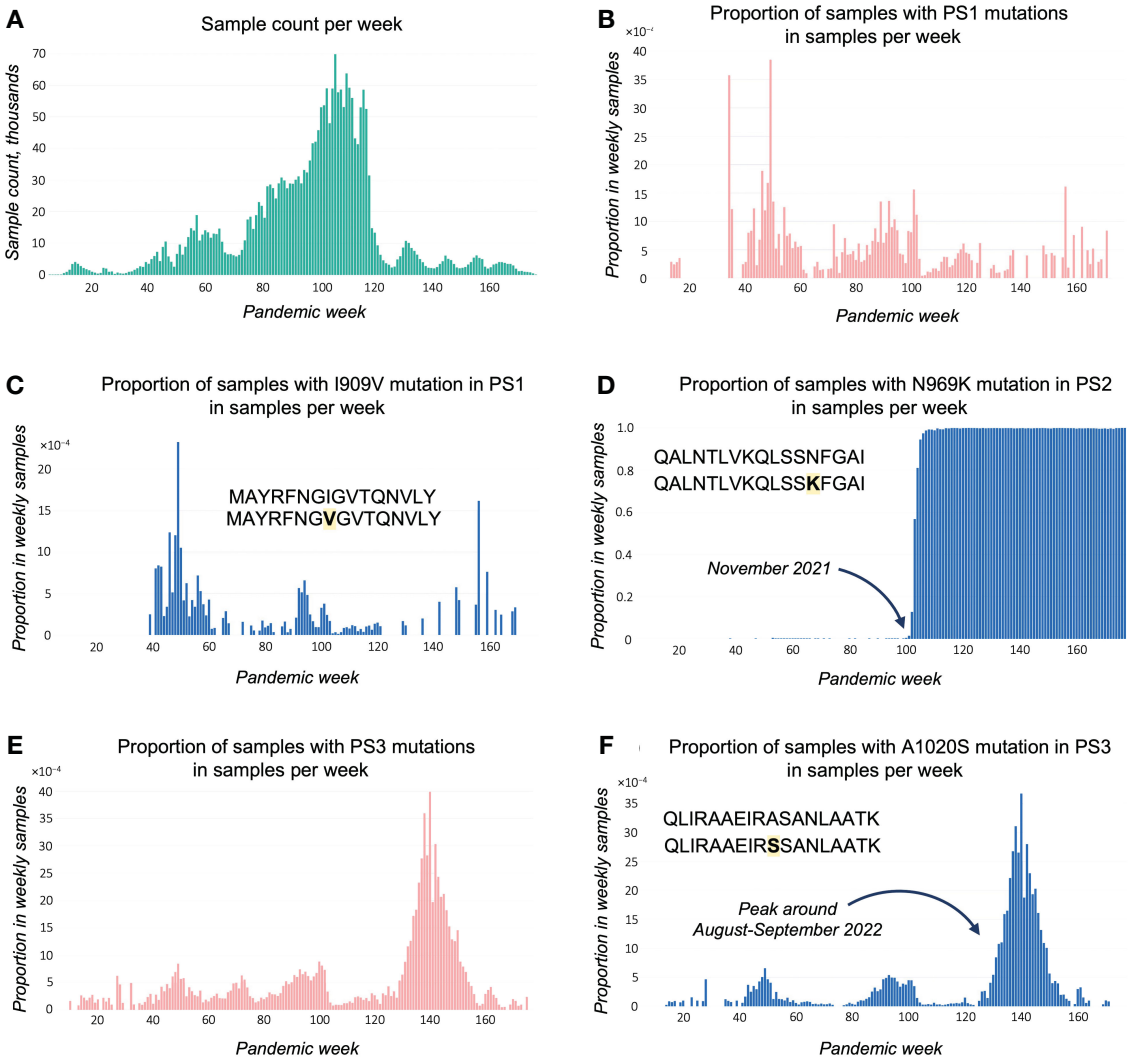
Time plots exported from EpitopeScan GUI. **(A)** Sample count per week for analyzed samples with metadata. Plots **(B-F)** show proportion (bound between 0 and 1) in weekly sample count for samples with **(B)** PS1 amino acid (AA) mutations; **(C)** PS1 I909V mutation; **(D)** PS2 N969K mutation; **(E)** PS3 AA mutations; **(F)** PS3 A1020S mutation. Proportions at plots **(C, D, F)** are calculated from samples with sufficient coverage and no disruptions in Spike protein open reading frame.

Finally, EpitopeScan GUI time plots provide a detailed picture of mutation spread in population (**Figure 3**). **Figure 3A** presents a context of weekly number of samples with metadata. The rest of the plots are normalized and provided for proportions of samples in weekly number of samples. Time plots for general statistics demonstrate that non-functional and insufficient coverage samples are rather evenly distributed throughout the pandemic (plots not shown), so that the data have no significant local abnormalities in terms of sampling and quality.

**Figure 3B** demonstrates that the proportion of samples with mutations in PS1 remained low (<0.4%) and fluctuated randomly throughout the pandemic. This pattern is closely resembled by the plot for the most abundant PS1 mutation I909V (**Figure 3C**), whereas mutations with lower counts have only minor contribution to the cumulative mutation illustrated in **Figure 3B**. A conservation plot for the PS1 sequence indicates that there is no bias in mutation toward any particular position or region of the peptide (**Supplementary Figure 4A**). These data conclude that the PS1 epitope is well conserved.

For PS2, the proportion of samples with mutations increases rapidly around November 2021 bordering to 1.0 (**Supplementary Figure 5A**). The plot for the N969K mutation (caused by A24469T substitution, **Supplementary Figure 4B**) completely repeats this trend (**Figure 3D**), showing that this mutation is the main cause of the loss of the PS2 epitope. This loss might represent an immune escape mechanism: N969K might affect binding to HLA-DRB1*04:01 and/or the recognition by PS2-specific CD4^+^ T cells. As detailed by a EpitopeScan GUI report, the mutation emerged with the first Omicron lineage BA.1 sticking with the following Omicron lineages. Therefore, HLA-DRB1*04:01 tetramer technology could be applied to detect variant-specific CD4^+^ T-cell responses using PS2 and PS2^N969K^.

In order to assess binding to the MHC II allele HLA-DRB1*04:01, a binding prediction was generated with the IEDB consensus tool for PS1–3 and PS2^N969K^ (**Supplementary Table 5**). Even though distinct algorithms, and the consensus prediction agree that PS2^N969K^ binding affinity to MHC II will be lower than PS2, it is unclear whether PS2^N969K^ has biding capability comparable with other peptides (as suggested by the SMM align component) or is in fact a worse binder than PS1–3 (as suggested by the NN align component).

Other PS2 mutations, Q957L and T961M, have peaks in occurrence around weeks 60–65 (February–March 2021) with low presence up to 0.2% and 0.8%, respectively (**Supplementary Figure 5B**). In addition, lineage statistics in EpitopeScan GUI show that these mutations are not defining any particular lineage. Therefore, these mutations along with the rest of PS2 mutations do not contribute significantly to the loss of the PS2 epitope.

An evident peak of samples with PS3 mutations appears around the 140th week (28/08/2022–03/09/2022, **Figure 3E**). This peak is caused by the most abundant PS3 mutation A1020S (**Table 3**), which is verified by the same spike at A1020S plot (**Figure 3F**). Lineage statistics from EpitopeScan GUI detail that A1020S is characteristic to Omicron BF.5 and BF.5.1 lineages being present in all samples assigned to these lineages. WSI lineage resource confirms that these lineages had been spreading in August–September 2022 with BF.5 being the major one. BF.5 demonstrates local bursts in England with up to 50%–60% cases in some regions. The overall maximum in total England’s samples is 3.4% (coherent with A1020S plot). The other abundant PS3 mutation A1020V displays random mutation history with low presence <1% (**Supplementary Figure 5C**) as well as other PS3 mutations with lower counts. Despite numerous low-count mutations being discovered for PS3, the conservation plot suggests that these minor mutations are evenly distributed around the PS3 region (**Supplementary Figure 4C**). Therefore, apart from the episode of BF.5 spread, PS3 appears to be rather well-conserved during the pandemic.

## 4 Discussion

Bioinformatic analysis of mutations emerging in SARS-CoV-2 epitopes is an informative aid to immunity research. In this work, we developed the EpitopeScan Python3 package to provide a user-friendly cross-platform framework facilitating a detailed analysis of mutation patterns in known SARS-CoV-2 antigenic peptides. With the combination of command-line and graphical interface tools, the package provides convenient overview of general sample statistics (such as proportion of non-functional samples, samples with insufficient coverage and no mutations) in time, of overall sequence conservation, as well as how distinct mutations emerged and propagated in population with respect to timeline and circulating lineages. The mutation data collected and summarized by EpitopeScan can be used to make decisions on applications of immunogenic peptides’ and to direct further investigation of their activity.

The only data inputs required by the EpitopeScan are the aligned genome sequences and a list of peptides to investigate for mutations. It is important to mind that the current version of EpitopeScan monitors for substitutions, deletions, and frameshifts but does not account for insertions. The latter limitation was accepted as no genomes in COG-UK data contain insertions, suggesting that such cases are negligible in SARS-CoV-2 mutation. While the discussed application case focuses on Spike protein peptides, EpitopeScan is in fact designed to analyze peptides originating from any SARS-CoV-2 protein. This report mainly focuses on linear peptide epitopes; however, EpitopeScan can be applied to conformational epitopes by passing multiple sequences forming a conformational epitope of interest. Finally, it is important to note that the current version of EpitopeScan is only suitable to handle SARS-CoV-2 sequences; however, the design of the package will allow to extend its functionality to other viruses in future versions.

We demonstrate the utility of the EpitopeScan workflow by investigating three S protein-derived peptides PS1–3 with the aim to suggest whether these peptides are suitable for immune monitoring of CD4^+^ T-cell responses with tetramer technology using samples collected in England from patients bearing the HLA-DRB1*04:01 allele. EpitopeScan tools allowed to detail diverse mutation patterns in peptides PS1–3. PS1 appears to be consistently conserved and also the most conserved one among the studied panel. Therefore, it can be loaded on a fluorophore-labelled HLA-DRB1*04:01 tetramer to detect an immune response in samples collected at any time point. In contrast, PS2 is the least conserved peptide featuring an N969K mutation characteristic to Omicron lineages (29), which were prevalent in England since around November 2021. Due to this mutation, tetramers loaded with PS2 are likely to be incapable of staining SARS-CoV-2-specific CD4^+^ T cells originating from infections with an Omicron variant. PS2^N969K^ could be used to achieve that; however, MHC II binding models predict PS2^N969K^ to have poorer binding to MHC II HLA-DRB1*04:01 than PS2. Although *in silico* estimation is insufficient to conclude whether PS2^N969K^ should be expected to completely lose HLA-DRB1*04:01 binding. Despite good performance of existing models in MHC binding prediction (30), estimates like these should be treated with caution as accurate universal predictions are unlikely due to the complicated nature of MHC-peptide binding. Experimental determination of peptide binding to HLA-DRB1*04:01 is necessary, and the applicability of PS2^N969K^-loaded HLA-DRB1*04:01 tetramers to profile Omicron-specific CD4^+^ T-cell responses is yet to be tested experimentally. Finally, PS3 features a peak of A1020S mutation linked to BF.5 Omicron lineage spread. Several regions in England were significantly affected by this lineage in August–September 2022 with up to 50%–60% of reginal genome samples corresponding to this lineage (23). Even though PS3 appears to be generally well-conserved, its reliability as a probe for immune monitoring is compromised because of the significant episode of A1020S spread.

Overall, EpitopeScan efficiently facilitates understanding of mutation within known SARS-CoV-2 antigenic peptides, which is valuable for the investigation of immunity and peptide’s application for immune monitoring. It should be noted that the current version of EpitopeScan is not particularly suited for general conservation screening of large peptide sets as, for example, done by Tarke et al. (31). Another shortfall of the package is that handling of large data frames leads to a significant RAM usage rapidly increasing with the number of input peptides and posing certain requirements on technical characteristics of the used hardware. Algorithm optimization and expansion of functionality to other viruses and insertion handling are the routes for development of next package versions.

## Data availability statement

EpitopeScan package with supporting manual is available via public GitHub repository (https://github.com/Aleksandr-biochem/EpitopeScan). The COG-UK genome MSA and metadata used in this work can be found in the achieved website of COG-UK project (https://webarchive.nationalarchives.gov.uk/ukgwa/20230507102210/https://www.cogconsortium.uk/priority-areas/data-linkage-analysis/public-data-analysis/).

## Ethics statement

The studies involving humans were approved by COVID-19 Genomics UK (COG-UK) consortium. The studies were conducted in accordance with the local legislation and institutional requirements. Written informed consent for participation was not required from the participants or the participants’ legal guardians/next of kin in accordance with the national legislation and institutional requirements.

## Author contributions

AK: Formal analysis, Software, Writing – original draft, Writing – review & editing. SV: Conceptualization, Formal analysis, Supervision, Writing – original draft, Writing – review & editing.
