# Supplementary material for: Development and application of EpitopeScan, a Python3 toolset for mutation tracking in SARS-CoV-2 immunogenic epitopes
Source: Front Immunol. 2024 May 22;15:1356314. doi: 10.3389/fimmu.2024.1356314 (PMC11150570; doi:10.3389/fimmu.2024.1356314)
Supplement: Supplementary file 2 [file DataSheet_2.docx]

Supplementary Material

# Supplementary Data

Supplementary Data is supplied as supplementary_data.zip archive and contains the following items:

- “README.txt” – a brief description of all the files in the archive;
- “Generate_test_sequences.ipynb” – Python3 jupyter notebook containing the code used to simulate test genomes and mutation data for EpitopeScan;
- “Verify_EpitopeScan_against_COGUK.ipynb” – Python3 jupyter notebook detailing comparison between EpitopeScan analysis of mutation in 3 Spike protein-derived epitopes based on COG-UK genome data and the mutation metadata from COG-UK;
- “performance_testing” – folder with materials for EpitopeScan performance testing, which contains:
  - “varying_l.sh”, bash script launching and timing EpitopesScan runs on the peptides of varying length;
  - “varying_n.sh”, bash script launching and timing EpitopesScan runs on varying number of peptides;
  - “varying_n”, folder with FASTA files for each run of EpitopeScan with a varying number of input peptides;
  - “run_time.xlsx”, Excel table with EpitopeScan run times recorded during the initial performance testing + constructed plots;

In addition, “EpitopeScan_outputs_for_PS1_3.zip” – an archive with EpitopeScan outputs generated for 3 Spike protein derived epitopes PS1-3 from England samples of COG-UK genome data. This data was generated specifying ambiguity intolerance `--ambiguity_threshold 0.0`. The folder contains 3 subfolders with output tables for each epitope.

# Supplementary Figures and Tables

## Supplementary Figures

**
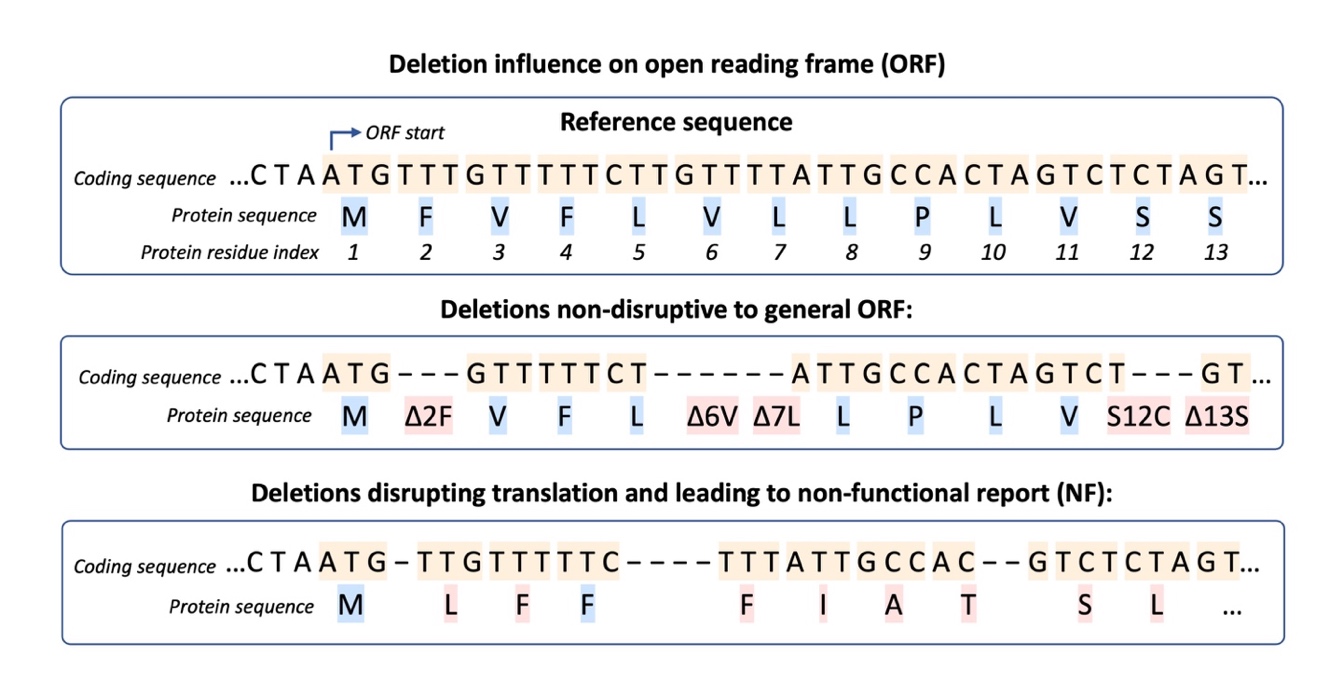
**

**Supplementary Figure 1.** Disrupting and non-disrupting deletions distinguished in EpitopeScan. Top panel provides an example of an Open Reading Frame (ORF) sequence (peach) and a translated peptide (light blue). Middle panel illustrates deletions, which do not disrupt translation frame as a whole. Bottom panel illustrates how deletions with number of positions unequal to a multiple of 3 lead to disrupted translation and loss of protein function.

**Supplementary Figure 2.** Input peptide length influence on EpitopeScan run time. Data points from table PS2. Trend line is an exponential function. Equation for the fitted model and R2 metric are displayed at the plot. Generated with Excel.

|   **Supplementary Figure 3.** (A) Influence of the number of input peptides (of fixed length 5) on EpitopeScan run time. (B) Influence of the Open Reading Frames cumulative length of input peptides parent proteins on EpitopeScan runtime. Data points from table PS3. Trend lines are exponential functions. The fitted models’ equations and R2 metrics are displayed at the plot. Generated with Excel. |
| --- |


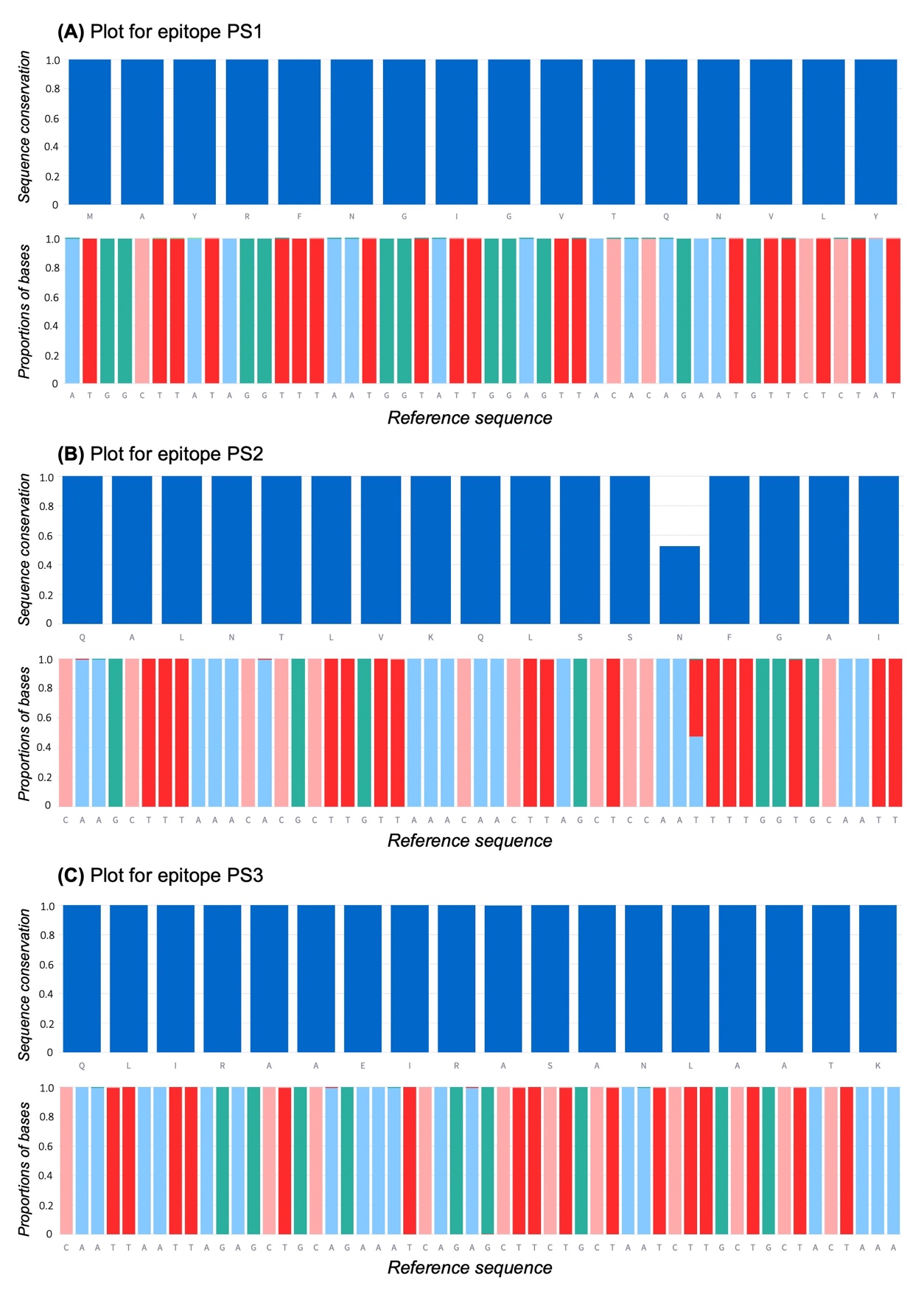


**Supplementary Figure 4.** Sequence conservation plots exported from EpitopeScan GUI. Plots A-C detail conservation for PS1-3 respectively. At each plot, upper subplot (blue) details peptide sequence conservation with reference sequence displayed along the x axis and the bars demonstrating proportion of all samples with the reference residue at this position (bound between 0 and 1). Lower subplots (colour) display conservation of reference coding sequence (displayed along the x axis). Stacked bar plot visualizes percentages of all bases at each position of the reference sequence. Proportions are calculated only from functional samples with sufficient coverage regardless of metadata presence.


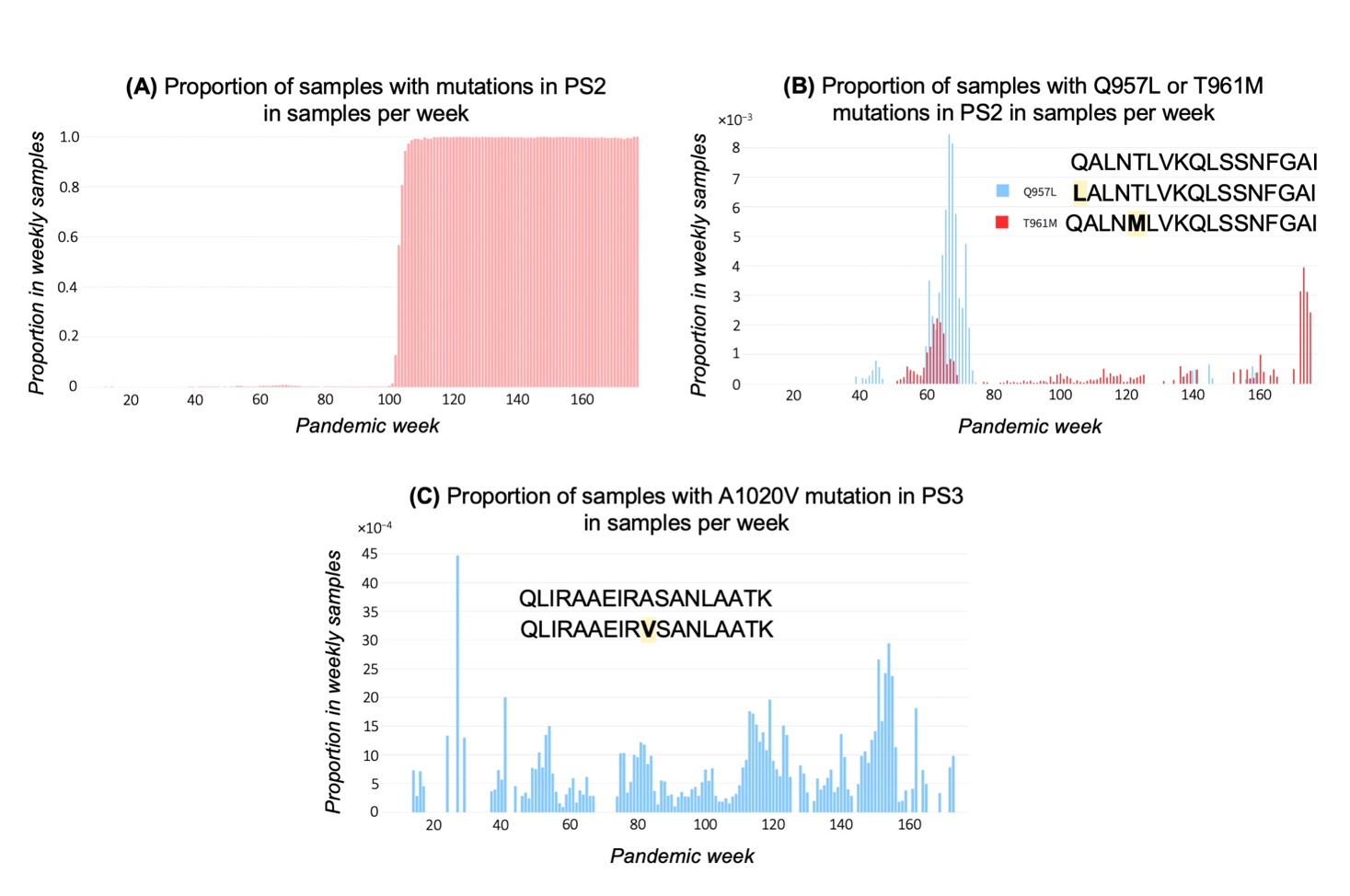


**Supplementary Figure 5.** Time plots exported from EpitopeScan GUI. Plots (A-C) show proportion in weekly sample count (bound between 0 and 1) for samples with: (A) PS2 amino acid (AA) mutations; (B) PS2 Q957L (blue) or T961M (red) mutation; (C) PS3 A1020V mutation. Proportions at plots (B,C) are calculated from samples with coverage and no Spike protein open reading frame disruptions.

## Supplementary Tables

Supplementary Table 1. List of test peptides with simulated genomes and reference mutation data supplied for EpitopeScan testing. Information listed for each peptide includes: peptide name, amino acid sequence, list of parent proteins, start and end residues in the primary parent protein (first one in a corresponding parent protein list), start and end coordinates in the reference genome. Peptide and genome indexing start from 1.

| **Peptide name** | **Peptide sequence** | **Parent protein** | **Protein start and end residues** | **Genome start and end coordinates** |
| --- | --- | --- | --- | --- |
| T1_Plp1ab | AGSYKDWSYSGQSTQLGIE | Plp1ab, Plp1a, NSP3 | 1508 – 1526 | 4787 – 4843 |
| T2_NSP12 | QSFLNRVCGVSAARL | Plp1ab, NSP12 | 4397 – 4411 | 13454 – 13497 |
| T3_Plp1ab | LTSHTVMPLSAPTLVPQEHY | Plp1ab, NSP13 | 5551 – 5570 | 16915 – 16974 |
| T4_Plp1a | SSEYKGPITDVFYKENSYT | Plp1ab, Plp1a, NSP3 | 1856 – 1874 | 5831 – 5887 |
| T5_S | RGVYYPDKVF | S | 34 – 43 | 21662 – 21691 |
| T6_NPS3a | RLWLCWKCRSKNPLLY | NPS3a, NPS3b | 126 – 141 | 25768 – 25815 |
| T7_NPS3b | FTSDYYQLYSTQLS | NPS3a, NPS3b | 207 – 220 | 26011 – 26052 |
| T8_E | NIVNVSLVKPSF | E | 45 – 56 | 26377 – 26412 |
| T9_M | INWITGGIAIAMAC | M | 73 – 86 | 26739 – 26780 |
| T10_NS6 | NKYSQLDEEQPMEI | NS6 | 47 – 60 | 27340 – 27381 |
| T11_NS7a | PIFLIVAAIVFITLCFTLKR | NS7a | 99 – 118 | 27688 – 27747 |
| T12_NS7b | LIIFWFSLELQDHNETCHA | NS7b | 25 – 43 | 27828 – 27884 |
| T13_NS8 | SKSPIQYIDIGNYTV | NS8 | 67 – 81 | 28092 – 28136 |
| T14_N | MSGKGQQQQGQTVT | N | 234 – 247 | 28973 – 29014 |
| T15_NS9a | NMARKTLNSLEDKAFQL | NS9a | 55 – 71 | 28446 – 28496 |
| T16_NS9b | ELLLLEWLAMA | NS9b | 51 – 61 | 28884 – 28916 |
| T17_NPS10 | RNYIAQVDVVNF | NPS10 | 24 – 35 | 29627 – 29662 |
| T18_NSP1 | LSLPVLQVRDVLVR | Plp1ab, Plp1a, NSP1 | 16 – 29 | 311 – 352 |
| T19_NSP2 | LNLGETFVTHS | Plp1ab, Plp1a, NSP2 | 703 – 713 | 2372 – 2404 |
| T20_NSP3 | VVDYGARFYFYTSKTT | Plp1ab, Plp1a, NSP3 | 1415 – 1430 | 4508 – 4555 |
| T21_NSP4 | IVAGGIVAIVVTCL | Plp1ab, Plp1a, NSP4 | 3047 – 3060 | 9404 – 9445 |
| T22_NSP5 | DFNLVAMKYNY | Plp1ab, Plp1a, NSP5 | 3492 – 3502 | 10739 – 10771 |
| T23_NSP6 | ILMTARTVYDDGARRVW | Plp1ab, Plp1a, NSP6 | 3693 – 3709 | 11342 – 11392 |
| T24_NSP7 | VLQQLRVESSSKL | Plp1ab, Plp1a, NSP7 | 3875 – 3887 | 11888 – 11926 |
| T25_NSP8 | VAKSEFDRDAAMQRKLEKM | Plp1ab, Plp1a, NSP8 | 3986 – 4004 | 12221 – 12277 |
| T26_NSP9 | NTTKGGRFVLALLSDLQDLK | Plp1ab, Plp1a, NSP9 | 4173 – 4192 | 12782 – 12841 |
| T27_NSP10 | PEANMDQESFG | Plp1ab, Plp1a, NSP10 | 4312 – 4322 | 13199 – 13231 |
| T28_NSP13 | IVVFDEISMATNYDLSVVN | Plp1ab, NSP13 | 5694 – 5712 | 17344 – 17400 |
| T29_NSP14 | EELFYSYATHSDKFTD | Plp1ab, NSP14 | 6289 – 6304 | 19129 – 19176 |
| T30_NSP15 | DLFRNARNGVLITEGS | Plp1ab, NSP15 | 6584 – 6599 | 20014 – 20061 |
| T31_NSP16 | CDLQNYGDSATLPKGIMMN | Plp1ab, NSP16 | 6823 – 6841 | 20731 – 20787 |

**Supplementary Table 2.** Influence of input peptide length on run time. Run time in minutes was recorded for EpitopeScan runs with default options. Runs completed on Spike protein peptides of varying length and 3,392,463 genomes samples across the UK.

| **S protein peptide length, residues** | **t, min** |
| --- | --- |
| 5 | 12.46 |
| 10 | 14.58 |
| 15 | 23.83 |
| 20 | 30.29 |
| 30 | 50.36 |
| 50 | 270.96 |

Supplementary Table 3. Influence of the number of input peptides on EpitopeScan run time. Run time in minutes recorded for runs with default options. Runs conducted on varying numbers of Spike protein peptides (fixed length 5) and 3,392,463 genomes samples across the UK. First column provides Open Reading Frames cumulative length of peptides’ parent proteins scanned in each sample for frame disruption control.

| **Cumulative ORFs length, bases** | **N peptides** | **t, min** |
| --- | --- | --- |
| 3821 | 1 | 12.13 |
| 4716 | 3 | 14.85 |
| 5382 | 6 | 19.59 |
| 26802 | 10 | 66.14 |
| 29767 | 20 | 89.12 |

Supplementary Table 4. Sample statistics for the analysis of PS1-3 from SRAS-CoV-2 Spike protein conducted with EpitopeScan under three configurations. For each peptide key information is listed: sequence, residues in S protein and genome coordinates. Modes listed in tables: (I) default options; (II) ambiguity threshold of 0.0 (presence of any ambiguous base in peptide coding region is treated as insufficient coverage); (III) ambiguity threshold of 0.0 and genome N content filter with upper threshold of 0.05 (5%). Runs conducted on 2,671,810 genomes sampled in England (accessed from COG-UK). For each samples’ category there is a count and a percentage in total number of analysed samples (does not include discarded samples). Statistics are calculated regardless of metadata availability for samples.

| *Peptide:* | **PS1** | | | | |
| --- | --- | --- | --- | --- | --- |
| *Sequence:* | MAYRFNGIGVTQNVLY | | | | |
| *S protein residues:* | 902 - 917 | | | | |
| *Genome coordinates:* | 24266 - 24313 | | | | |
| Mode | I | II | | III | |
| Have mutations | 1,291 (0.05 %) | 1,288 (0.05 %) | | 1,054 (0.05 %) | |
| No mutations | 2,603,579 (97.45 %) | 2,599,376 (97.29 %) | | 2,215,960 (99.6 %) | |
| No coverage | 57,527 (2.15 %) | 61,733 (2.31 %) | | 2,373 (0.11 %) | |
| Non-functional | 9,413 (0.35 %) | 9,413 (0.35 %) | | 5,429 (0.24 %) | |
| Discarded by quality | 0 | 0 | | 446,994 | |
|  |  | |  | |  |
| *Peptide:* | **PS2** | | | | |
| *Sequence:* | QALNTLVKQLSSNFGAI | | | | |
| *S protein residues:* | 957-973 | | | | |
| *Genome coordinates:* | 24431-24481 | | | | |
| Mode | I | II | | III | |
| Have mutations | 1,249,720 (46.77 %) | 1,245,269 (46.61 %) | | 1,016,768 (45.70 %) | |
| No mutations | 1,378,532 (51.60 %) | 1,375,921 (51.50 %) | | 1,200,858 (53.98 %) | |
| No coverage | 34,145 (1.28 %) | 41,207 (1.54 %) | | 1,761 (0.08 %) | |
| Non-functional | 9,413 (0.35 %) | 9,413 (0.35 %) | | 5,429 (0.24 %) | |
| Discarded by quality | 0 | 0 | | 446,994 | |
|  |  | |  | |  |
| *Peptide:* | **PS3** | | | | |
| *Sequence:* | QLIRAAEIRASANLAATK | | | | |
| *S protein residues:* | 1011-1028 | | | | |
| *Genome coordinates:* | 24593-24646 | | | | |
| Mode | I | II | | III | |
| Have mutations | 10,517 (0.39 %) | 10,498 (0.39 %) | | 8,791 (0.40 %) | |
| No mutations | 2,610,586 (97.71 %) | 2,605,459 (97.52 %) | | 2,209,307 (99.30 %) | |
| No coverage | 41,294 (1.55 %) | 46,440 (1.74 %) | | 1,289 (0.06 %) | |
| Non-functional | 9,413 (0.35 %) | 9,413 (0.35 %) | | 5,429 (0.24 %) | |
| Discarded by quality | 0 | 0 | | 446,994 | |

Supplementary Table 5. IEDB MHC II prediction results for peptides PS1-3 and the PS2 peptide woth N969K mutation. Calculated with T cell epitope MHC II binding prediction online tool (IEDB Analysis Resource v2.26). Columns of the table with 5 rows are wrapped on several lines.

| **Peptide** | **Sequence** | **Percentile Rank** | **Adjusted Rank** |
| --- | --- | --- | --- |
| PS2 | QALNTLVKQLSSNFGAI | 1.7 | 2.9 |
| PS3 | QLIRAAEIRASANLAATK | 2.5 | 7.4 |
| PS2^N969K^ | QALNTLVKQLSSKFGAI | 9.3 | 15.84 |
| PS1 | MAYRFNGIGVTQNVLY | 26 | 30.01 |
|  |  |  |  |
| **SMM-align core** | **SMM-align IC50** | **SMM-align rank** | **SMM-align adjusted rank** |
| LVKQLSSNF | 230 | 1.7 | 2.9 |
| IRASANLAA | 381 | 5 | 14.8 |
| LVKQLSSKF | 347 | 3.8 | 6.47 |
| FNGIGVTQN | 2137 | 37 | 42.71 |
|  |  |  |  |
| **NN-align core** | **NN-align IC50** | **NN-align rank** | **NN-align adjusted rank** |
| VKQLSSNFG | 46.6 | 0.64 | 1.09 |
| IRASANLAA | 104.5 | 2.5 | 7.4 |
| LVKQLSSKF | 310.2 | 9.3 | 15.84 |
| FNGIGVTQN | 101.5 | 3.2 | 3.69 |
|  |  |  |  |
| **Sturniolo core** | **Sturniolo score** | **Sturniolo rank** | **Sturniolo adjusted rank** |
| LVKQLSSNF | 1.7 | 22 | 37.47 |
| IRASANLAA | 4 | 2.1 | 6.22 |
| LVKQLSSKF | 1.7 | 22 | 37.47 |
| YRFNGIGVT | 1.3 | 26 | 30.01 |
